# Psychosocial Constructs and Postintervention Changes in Physical Activity and Dietary Outcomes in a Lifestyle Intervention, Hub City Steps, 2010

**DOI:** 10.5888/pcd12.140525

**Published:** 2015-05-21

**Authors:** Alicia S. Landry, Jessica L. Thomson, Michael B. Madson, Jamie M. Zoellner, Richard S. Mohn, Jeremy Noble, Carol L. Connell, Kathy Yadrick

**Affiliations:** Author Affiliations: Jessica L. Thomson, USDA Agricultural Research Service, Baton Rouge, Louisiana; Michael B. Madson, Jeremy Noble, The University of Southern Mississippi, Department of Psychology, Hattiesburg, Mississippi; Jamie M. Zoellner, Virginia Polytechnic Institute and State University, Department of Human Nutrition, Foods and Exercise, Blacksburg, Virginia; Richard S. Mohn, The University of Southern Mississippi, Department of Educational Studies and Research, Hattiesburg, Mississippi; Carol L. Connell, Kathy Yadrick, The University of Southern Mississippi, Department of Nutrition and Food Systems, Hattiesburg, Mississippi.

## Abstract

**Introduction:**

Although modifications to dietary and physical activity (PA) behavior can reduce blood pressure, racial disparities in prevalence and control of hypertension persist. Psychosocial constructs (PSCs) of self-regulation, processes of change, and social support are associated with initiation and maintenance of PA in African Americans; which PSCs best predict lifestyle behavior changes is unclear. This study’s objective was to examine relationships among PSC changes and postintervention changes in PA and dietary outcomes in a community-based, multicomponent lifestyle intervention.

**Methods:**

This study was a noncontrolled, pre/post experimental intervention conducted in a midsized, Southern US city in 2010. Primarily African American adults (n = 269) participated in a 6-month intervention consisting of motivational enhancement, social support, pedometer diary self-monitoring, and 5 education sessions. Outcome measures included pedometer-determined steps per day, fitness, dietary intake, and PSC measures. Generalized linear mixed models were used to test for postintervention changes in behavioral outcomes, identify predictors of PSC changes, and determine if PSC changes predicted changes in PA and diet.

**Results:**

Postintervention changes were apparent for 10 of 24 PSCs (*P* < .05). Processes of change components, including helping relationships, reinforcement management, and consciousness raising, were significant predictors of fitness change (*P* < .05).

**Conclusion:**

This article is among the first to address how measures of several theoretical frameworks of behavior change influence changes in PA and dietary outcomes in a multicomponent, community-based, lifestyle intervention conducted with African American adults. Findings reported identify PSC factors on which health behavior interventions can focus.

## Introduction

Despite increased awareness of its associated health risks, US prevalence of hypertension remained consistent (30%) during the past 10 years (1). Likewise, persistent disparities are apparent among African Americans, whose prevalence is higher (41%) than that of non-Hispanic whites (29%) (1). Furthermore, racial disparities in hypertension control exist; 43% of African Americans have controlled hypertension, whereas 53% of non-Hispanic whites do (1). Although pharmacologic treatment is commonly prescribed to control hypertension, modifications to diet and physical activity (PA) lifestyle can effectively reduce blood pressure (BP), thereby controlling hypertension and reducing cardiovascular event risks. Theoretically based, diet- and PA-related lifestyle interventions are effective at reducing BP in African American adults (2). However, mechanisms resulting in or leading to behavioral changes are poorly understood, although several theories and constructs have been proposed (ie, Transtheoretical Model, self-determination theory, and social support); in studies where psychosocial constructs (PSCs) are evaluated, results are often inconsistent.

Using a polytheoretical approach incorporating key constructs from the Transtheoretical Model (3), SDT (4,5), and social support frameworks (6,7), we designed and conducted HUB (A Healthy “U” Begins with Steps) City Steps, a behavioral lifestyle intervention, in a Southern, primarily African American cohort in 2010. We previously published the positive effects of this study’s primary outcome, improving BP (8,9) and demonstrated significant improvements in step-determined PA and sugar intake, but not in body mass index (BMI) (10). However, associations between changes in PSCs and health outcomes have yet to be explored. Given the successful effects on BP and the lack of investigation of specific processes of change, we sought to better appreciate how changes in processes of change were predictive of changes in PA and diet. A secondary objective was determining sociodemographic predictors of PSC changes.

## Methods

### Design and sample

HUB City Steps was a 2-phase, community-based, lifestyle intervention with multiple components that targeted hypertension risk factors. The first phase was a 6-month noncontrolled, pre/post experimental intervention that was conducted from the end of January to the beginning of August 2010 and is the focus of this article. The second phase consisted of a 12-month maintenance intervention designed to test treatment effects of participants, who were randomized to a low versus high (ie, 4 vs 10) dose of telephone-delivered sessions that used a motivational interviewing approach. A full description of the methods has been published (8,11). All phases of this research were approved by the University of Southern Mississippi’s institutional review board.

Recruitment efforts primarily targeted African American residents in Hattiesburg, a midsized city of nearly 46,000 in southeast Mississippi, where approximately 53% of residents are African American and 42% are white (12). Eligibility criteria included adults aged 18 years or older who were English-speaking, were noninstitutionalized, and resided in the Hattiesburg area. Participants with systolic/diastolic BP of 180/110 mm Hg or higher were directed to obtain immediate medical attention and were disqualified from participation in the study. All others were eligible for participation regardless of BP and hypertension medication regimen. Informed consent and signed medical disclaimer were obtained upon enrollment into the study.

### Measures and intervention

Various PSC, PA, and dietary measures were assessed. The interviewer-administered PSC instruments included measures of self-determination theory (treatment self-regulation for PA and for diet; 15 items each, 4 subscales: amotivation, external regulation, introjection, identification, and integration; score range, 30–150 [13]), processes of change for PA (30 items, 10 subscales: stimulus control, social liberation, reinforcement management, helping relationships, counter conditioning, self-liberation, self-reevaluation, environmental reevaluation, dramatic relief, and consciousness raising; score range, 30–150 [14]), and social support from walking group (11 items, 3 subscales: guidance, reliable alliance, and reassurance of worth) and from coach for PA (12 items, 3 subscales: guidance, reliable alliance, and social integration; score range for both, 23–115 [15]). The 6-minute walk test, an objective, simple, inexpensive, and safe exercise test (16,17), was used as a measure of fitness. The 6-minute walk test is reliable and can discriminate between functional levels in a high-functioning population (18). Physical activity was measured using Yamax pedometers (HRM USA, Inc). Each participant received a pedometer at baseline assessment with stride calibration determined by the 6-minute walk test. Participants had the option of recording daily steps on weekly pedometer diary postcards or by logging on to the intervention’s website. The National Cancer Institute’s 5-Factor Screener was used to assess dietary intake. This valid 18-item screener approximates intakes of fruit, vegetable, and dairy (servings); fiber (g); added sugar (tsp); and calcium (mg) (19,20).

The 6-month active intervention phase included 3 motivational enhancement sessions provided by intervention staff, continuous social support provided by walking coaches and walking groups, weekly pedometer diary self-monitoring, and 5 monthly education sessions (8,9,21). The motivational enhancement sessions implemented were consistent with self-determination theory and processes of change and focused on building internal motivation for change. During motivational enhancement sessions, participants received personalized feedback about various health factors such as weight, BP, cholesterol, BMI (weight in kilograms divided by the square of height in meters), and diet. Participants were given the opportunity to choose which health topics they wanted to discuss with a health coach trained in motivational interviewing (21). Education sessions were approximately 90 minutes; 15 minutes were allotted for group PA, followed by 30 to 45 minutes of nutrition education congruent with Dietary Approaches to Stop Hypertension (22), and 30 minutes of social support enhancement through sharing of successes and challenges. Each session was developed to increase knowledge and facilitate behavior changes by promoting and supporting processes of change.

### Analysis

Statistical analyses were performed using SAS software, versions 9.3 and 9.4 (SAS Institute, Inc). Descriptive measures were used to summarize demographic characteristics, PSCs, and outcome variables. For the PSC scales, Cronbach’s α values were computed as a measure of internal consistency. According to guidance proposed by George and Mallery (23), values greater than or equal to 0.6 indicated acceptable internal consistency for these scales.

For modeling purposes, race was categorized as African American or other (including white and American Indian/Alaska native), marital status was categorized as married or not married (including widowed, divorced, separated, and never married), education was categorized as less than or equal to high school graduate or greater than high school graduate, and income was treated as a continuous variable because of the large number (n = 12) of original categories. Generalized linear mixed models, using maximum likelihood estimation, were used to test for significant time differences in outcomes and PSCs. Maximum likelihood estimation is an approach for handling missing data in repeated measures (24). Time (at baseline and at 3 and 6 months) was modeled as a repeated measure by using a first-order autoregressive covariance structure. Custom contrasts were used to test for significant differences between baseline and 3-month follow-up and between baseline and 6 months follow-up using a Bonferroni correction factor to account for multiple testing.

Generalized linear mixed models also were used to determine significant demographic characteristics and baseline values that predicted changes in PSCs, and to determine whether PSC changes predicted changes in PA and dietary outcomes while accounting for demographic characteristics and baseline values. Five models based on theoretical groupings identified in the literature were built for PA and dietary outcomes. For these models, PSC variables with Cronbach’s α values of less than 0.6 were excluded because of their potential unreliability in this cohort of participants.

Changes between baseline and 3 months and baseline and 6 months were modeled as repeated measures by using a variance component covariance structure. Least squares means were computed to estimate and compare outcome changes. Although we used change in pedometer-determined mean steps per day as a PA outcome, the change was not from baseline to 6 months because no data on baseline steps per day were collected. Therefore, change was calculated as the difference between steps per day reported during the first 2 weeks of the intervention and the remaining weeks (weeks 3–27). Positive changes represent an increase in steps per day between the initial and remaining weeks for the intervention period. Therefore, change in intervention steps per day represents persistence in or maintenance of step-defined PA rather than a true change from a preintervention baseline. Details about the rationale, methods, and use of this steps-per-day indicator can be found elsewhere (10). The significance level of the tests was set at .05 (.025 for multiple comparisons).

## Results

### Sample characteristics

Most participants were African American (94%) and female (85%); mean age of participants was 44 years (Table 1). Less than half (42%) were married, more than three-fourths (80%) had some post-high school education, and 27% reported a household income greater than or equal to $50,000 per year. Mean BMI was 35 kg/m^2^ (range, 17–64 kg/m^2^), and mean BP was 126/83 mm Hg.

**Table 1:** Baseline Characteristics of Participants (N = 269), HUB City Steps, 2010^a^

Characteristic	Value
**Sex**	
Male	40 (14.9)
Female	229 (85.1)
**Race**	
African American	254 (94.4)
Other^b^	15 (5.6)
**Marital status**	
Married	113 (42.0)
Not married^c^	156 (58.0)
**Education**	
≤High school graduate	53 (19.7)
>High school graduate	216 (80.3)
**Household income^d^, $**	
<10,000	40 (14.9)
10,000–19,999	36 (13.4)
20,000–29,999	54 (20.1)
30,000–39,999	37 (13.8)
40,000–49,999	30 (11.2)
≥50,000	71 (26.5)
**Current smoker**	23 (8.6)
**Diagnosed high blood pressure**	113 (42.0)
**Diagnosed high blood glucose**	42 (15.6)
**Diagnosed high cholesterol**	52 (19.3)
**Mean age, y (SD)**	44.3 (12.2)
**Mean systolic blood pressure, mm Hg (SD)**	126.0 (19.1)
**Mean diastolic blood pressure, mm Hg (SD)**	83.2 (12.3)
**Mean waist circumference, cm (SD)**	102.1 (18.1)
**Mean body mass index, kg/m^2^ (SD)**	34.7 (8.1)

Abbreviation: SD, standard deviation.

a Values are expressed as number (%), unless otherwise indicated.

b Includes white and American Indian/Alaska Native.

c Includes widowed, divorced, separated, and never married.

d Income denominator is 268 because of a missing response.

Of the 269 baseline participants, 227 (84%) were assessed at 3 months and 190 (71%) were assessed at 6 months follow-up. Comparisons between study noncompleters and completers at 3 months follow-up indicated that noncompleters were significantly younger (36 vs 46 years, *P* < .001) and had higher mean BMI (37 vs 34 kg/m^2^, *P* = .01), fat mass (48 vs 42 kg, *P* = .04), and lean body mass (57 vs 52 kg, *P* = .04) at baseline than completers (data not shown). Similarly, at 6 months follow-up, study noncompleters were significantly younger (39 vs 47 y, *P* < .001), had higher mean BMI (37 vs 34 kg/m^2^, *P* = .01), and lower mean triglycerides (117 vs 137 mg/dL, *P* = .04) at baseline than completers (data not shown).

### Assessment of psychosocial construct reliability and changes in outcome measures

Using baseline data, most (21 of 24) of the PSC scales and subscales demonstrated acceptable internal consistency, with Cronbach’s α at or above .60 (23) (Table 2). Unacceptable internal consistency was observed for 3 scales: amotivation for physical activity, dramatic relief, and self-liberation (Cronbach’s α range, 0.44–0.54). For the dietary and PA outcome variables, time differences were apparent only for sugar intake and steps per day. Sugar intake decreased by approximately 3 teaspoons at both follow-up times, while pedometer-determined PA increased by approximately 2,010 steps per day (Table 2).

**Table 2:** Mixed-Model Linear Regression Analyses for Time Differences in Study Outcomes, HUB City Steps, 2010

Outcome	No. of Items	Cronbach ɑ	Baseline	3 Months	6 Months	*P* Value^a^
			(N = 269)	(N = 227)	(N = 190)	
			Mean (Standard Deviation)			
**Dietary intake**						
Sugar, tsp	—	—	17.1 (9.0)	13.9 (7.0)	14.5 (7.7)	<.001
Calcium, mg	—	—	635 (421)	601 (355)	582 (322)	.30
Dairy, cups	—	—	1.0 (0.7)	1.0 (0.7)	1.0 (0.6)	.42
Fiber, g	—	—	14.1 (5.9)	13.9 (5.9)	14.0 (5.7)	.88
Fruits and vegetables, cups	—	—	2.6 (1.3)	2.6 (1.2)	2.6 (1.3)	.93
**Physical activity**						
Steps/day^b^	—	—	5,615.1 (2,766.8)	—	7,624.7 (4,226.9)	<.001
Fitness (6-min walk test)	—	—	440.0 (69.0)	452.0 (81.0)	449.0 (70.0)	.25
**Processes of change, behavioral^c^**						
Stimulus control	3	0.82	8.0 (3.6)	9.4 (3.4)	9.6 (3.2)	<.001
Helping relationships	3	0.91	9.5 (3.8)	11.0 (3.2)	11.1 (3.1)	<.001
Reinforcement management	3	0.70	12.2 (2.2)	12.7 (2.2)	12.7 (2.0)	.04
Counter-conditioning	3	0.82	7.9 (2.9)	9.6 (2.6)	9.9 (2.4)	<.001
Self-liberation	3	0.54	12.5 (1.9)	12.6 (1.8)	12.4 (1.8)	.40
**Processes of change, cognitive^c^**						
Consciousness-raising	3	0.83	9.3 (3.0)	9.7 (2.8)	9.9 (2.8)	.06
Dramatic relief	3	0.51	9.4 (2.4)	9.5 (2.5)	9.6 (2.7)	.48
Environmental reevaluation	3	0.66	12.3 (2.3)	12.4 (2.4)	12.5 (2.5)	.65
Social liberation	3	0.63	11.6 (5.3)	12.3 (2.1)	12.3 (2.1)	<.001
Self-reevaluation	3	0.73	13.6 (1.8)	13.8 (1.7)	13.6 (1.8)	.59
**Treatment self-regulation, diet^c^**						
Diet external regulation	4	0.85	9.2 (4.4)	8.9 (4.3)	8.8 (4.5)	.63
Diet introjected regulation	2	0.80	6.7 (2.6)	6.6 (2.7)	6.3 (2.6)	.30
Diet autonomous motivation	6	0.87	28.1 (2.8)	27.8 (2.9)	28.0 (2.8)	.59
Diet amotivation	3	0.62	6.2 (2.8)	5.5 (2.5)	5.3 (2.6)	.001
**Treatment self-regulation, physical activity^c^**						
PA external regulation	4	0.79	9.8 (4.2)	9.3 (3.9)	9.2 (4.1)	.26
PA introjected regulation	2	0.80	7.1 (2.3)	7.0 (2.4)	6.8 (2.4)	.30
PA autonomous motivation	6	0.82	27.7 (2.8)	27.6 (2.9)	27.5 (2.8)	.82
PA amotivation	3	0.44	6.6 (2.6)	6.0 (2.6)	5.9 (2.6)	.01
**Social support, coach^c^**						
Coach reliable alliance	3	0.77	13.0 (2.2)	13.3 (2.3)	13.3 (2.3)	.40
Coach guidance	4	0.78	16.5 (3.1)	16.9 (3.2)	16.8 (3.3)	.26
Coach reassurance of worth	4	0.79	16.3 (3.0)	17.3 (3.0)	17.4 (3.2)	<.001
**Social support, group^c^**						
Group reliable alliance	4	0.93	16.8 (3.5)	17.9 (2.9)	17.6 (3.0)	.001
Group guidance	4	0.91	16.3 (3.5)	17.2 (3.1)	17.1 (3.2)	.002
Group social integration	4	0.77	15.1 (3.0)	16.0 (3.1)	15.9 (3.1)	.002

Abbreviations: — , not assessed; PA, physical activity.

a *P* for time difference test; pair-wise comparisons (baseline to 3 months and baseline to 6 months) significant for all models with significant time effect except reinforcement management.

b Baseline value is mean of intervention weeks 1 and 2; 6 months value is mean of remaining intervention weeks (3–27). No 3-month value was computed.

c The interviewer-administered psychosocial instruments included measures of self-determination theory (treatment self-regulation for PA and for diet; 15 items each, 4 subscales: amotivation, external regulation, introjection, identification and integration; score range, 30–150 [13]), processes of change for PA (30 items, 10 subscales: stimulus control, social liberation, reinforcement management, helping relationships, counter conditioning, self-liberation, self-reevaluation, environmental reevaluation, dramatic relief, and consciousness raising; score range, 30–150 [14]), and social support from walking group (11 items, 3 subscales: guidance, reliable alliance, reassurance of worth) and from coach for PA (12 items, 3 subscales: guidance, reliable alliance, social integration; score range for both, 23–115 [15]). Mean (SD) values are scores.

Of the 21 scales with sufficient internal consistency, time differences were apparent for 10 of the constructs. At follow-up, scores for diet amotivation were significantly lower compared with baseline scores. Conversely, at 3 and 6 months follow-up, scores for coach reassurance of worth, group reliable alliance, group guidance, group social integration, counter-conditioning, helping relationships, social liberation, and stimulus control were significantly higher compared with baseline scores. For reinforcement management, pair-wise comparisons of 3 and 6 months scores to baseline failed to reach significance, although scores were higher at the follow-up times.

### Predictors of changes in psychosocial constructs

Only the 10 constructs with acceptable reliability and significant changes postintervention were included in mixed-model linear regression analyses for changes in PSCs. Sex was a significant predictor of change for diet amotivation (*P* < .05). A significant decrease was apparent for women, but the change was not significant for men. Marital status was a significant predictor of change for diet amotivation and stimulus control (*P* < .05). A significant decrease in diet amotivation was apparent for married participants; the significant increase in stimulus control observed for unmarried participants was higher than the significant increase for married participants (1.6 and 0.9 points, respectively). Smoking status was a significant predictor of change for social liberation; the change observed for smokers was higher than the significant increase for nonsmokers (1.2 and .5 points, respectively). Education level was a significant predictor of change for counter-conditioning, helping relationships, and reinforcement management (*P* < .05). For all 3 constructs, the significant increases observed for participants with less than or equal to a high school degree were greater than the significant increases for participants with more than a high school degree (*P* < .05). Age predicted change in counter-conditioning, baseline BMI predicted change in group reliable alliance, and both income and baseline BMI predicted change in stimulus control (*P* < .05).

### Changes in physical activity and dietary outcomes predicted by psychosocial construct changes

Results of the mixed-model linear regression analyses for changes in PA and dietary outcomes predicted by PSC changes are presented in Table 3 (all PSC and only significant covariates are reported). For behavioral processes of change, helping relationships and reinforcement management were significant predictors of fitness change. For constructs from self-determination theory, external regulation was a significant positive predictor of changes in both sugar and fiber intake; introjected regulation was a significant negative predictor of change in sugar intake. None of the treatment self-regulation constructs were significant predictors for fitness or intakes of calcium, dairy, and fruits and vegetables. For the cognitive processes of change, consciousness raising was a significant positive predictor of change in fitness. None of the processes of change were significant predictors of change in steps per day. For social support, group guidance was a significant negative predictor of change in steps per day. None of the social support constructs were significant predictors for change in fitness. For all 5 of the fitness models, the baseline fitness value was a significant negative predictor of change (regression coefficients = −.7). That is, participants who scored lower on the fitness test (ie, walked a shorter distance in 6 minutes) at baseline had greater improvements in fitness than did participants who scored higher at baseline. None of the covariates were significant predictors of change for the steps-per-day models. In terms of dietary outcomes, female participants significantly decreased their sugar intake by 3.7 teaspoons and their fiber intake by 0.6 grams, whereas male participants increased both by 2.6 teaspoons and 1.1 gram, respectively. Household income, baseline BMI, and baseline sugar intake were significant predictors of change in sugar intake. Similarly, baseline fiber intake was a significant predictor of change in fiber intake.

**Table 3:** Mixed-Model Linear Regression Analyses for Changes in Physical Activity and Dietary Outcomes Predicted by Psychosocial Construct Changes, HUB City Steps, 2010^a^

Psychosocial Constructs	Physical Activity				Diet^b^			
	Steps/d		Fitness		Sugar		Fiber	
	β	SE	β	SE	β	SE	β	SE
**Treatment self-regulation^c^**								
External regulation	NS	—	NS	—	0.2	0.08	0.2	0.07
Introjected regulation	NS	—	NS	—	−0.4	0.14	NS	
Motivation	NS	—	NS	—	NS		NS	
Amotivation	NA	—	NA	—	NS		NS	
Covariates^d^	NS	—	BOV	—	Sex, income, BMI, BOV		Sex, BOV	
**Processes of change, behavioral^c^**								
Stimulus control	NS	—	NS	—	—	—	—	—
Helping relationships	NS	—	−2.8	1.15	—	—	—	—
Reinforcement management	NS	—	3.6	1.81	—	—	—	—
Counter-conditioning	NS	—	NS	—	—	—	—	—
Covariates^d^	NS	—	BOV	—	—	—	—	—
**Processes of change, cognitive^c^**								
Consciousness raising	NS	—	4.0	1.54	—	—	—	—
Environmental reevaluation	NS	—	NS	—	—	—	—	—
Social liberation	NS	—	NS	—	—	—	—	—
Self-reevaluation	NS	—	NS	—	—	—	—	—
Covariates^d^	NS	—	BOV	—	—	—	—	—
**Social support, coach^c^**								
Guidance	NS	—	NS	—	—	—	—	—
Reliable alliance	NS	—	NS	—	—	—	—	—
Reassurance of worth	NS	—	NS	—	—	—	—	—
Covariates^d^	NS	—	BOV	—	—	—	—	—
**Social support, group^c^**								
Guidance	−153	75.7	NS	—	—	—	—	—
Reliable alliance	NS	—	NS	—	—	—	—	—
Social integration	NS	—	NS	—	—	—	—	—
Covariates^d^	NS	—	BOV	—	—	—	—	—

Abbreviations: —, not assessed; BMI, body mass index; BOV, baseline outcome value; NA, not applicable (unacceptable scale internal consistency); NS, nonsignificant; SE, standard error.

a Fitness changes = 3 months minus baseline and 6 months minus baseline; steps per day changes = intervention weeks 3–27 minus weeks 1–2. Nonsignificant variables were removed from the model.

b None of the psychosocial constructs were significant for the calcium, dairy, and fruit and vegetable models.

c The interviewer-administered psychosocial instruments included measures of self-determination theory (treatment self-regulation for PA and for diet; 15 items each, 4 subscales: amotivation, external regulation, introjection, identification and integration; score range, 30–150 [13]), processes of change for PA (30 items, 10 subscales: stimulus control, social liberation, reinforcement management, helping relationships, counter conditioning, self-liberation, self-reevaluation, environmental reevaluation, dramatic relief, and consciousness raising; score range, 30–150 [14]), and social support from walking group (11 items, 3 subscales: guidance, reliable alliance, reassurance of worth) and from coach for PA (12 items, 3 subscales: guidance, reliable alliance, social integration; score range for both, 23–115 [15]).

d Included time, age, sex, marital status, education, smoking status, income, baseline BMI, and baseline outcome value. Only significant covariates reported.

## Discussion

We found significant improvements in self-determination theory constructs of treatment self-regulation (diet amotivation), processes of change (counter-conditioning, helping relationships, reinforcement management, social liberation, and stimulus control), and social support (coach reassurance of worth, group reliable alliance, group guidance, and group social integration), as well as 2 behavioral improvements (pedometer-determined PA [steps/d] and dietary [sugar] intake). For treatment self-regulation constructs, only diet amotivation changed in the direction hypothesized, confirming results reported by others (25–27). We could find no studies in the literature supporting our finding of moderating effects of sex on amotivation in adults. However, we did find 1 study suggesting that in adolescents, sex plays a role in psychosocial processes leading to amotivation (28); therefore, invariance in amotivation may exist across multiple domains and populations that have yet to be investigated.

Postintervention amotivation change was not a significant predictor of postintervention changes in dietary outcomes. Increases in external regulation (ie, engaging in a behavior to satisfy external pressures or achieve external rewards) predicted increases in sugar and fiber intake, whereas an increase in introjected regulation (internalization of external controls to avoid guilt) predicted a decrease in sugar intake. These results suggest that the use of external rewards may be a useful method for increasing fiber intake but not for decreasing sugar intake, whereas the use of guilt may be a useful method for decreasing sugar intake in this population of African American adults. However, introjected regulation and guilt are not ideal forms of motivation because they foster anxiety and can make it difficult for people to feel positive and confident about their actions; thus, maintenance over time is unlikely (5).

HUB City Steps participants increased use of behavioral processes of change methods to a greater extent than they did cognitive methods. The increase in stimulus control was largest in unmarried participants and was positively associated with income and BMI. Single people may have more control over external or environmental stimuli than do married people, who need to accommodate spousal or family needs and desires. However, more research is needed to test this hypothesis. We found 1 study that reported sex differences in the use of stimulus control behaviors for weight loss (29) and no studies with marital-, income-, or BMI-related differences in the use of stimulus control for health behavior changes. Invariance in processes of change constructs applied to PA behavior was found between groups that differed in sex, age, and race/ethnicity (30). However, to our knowledge, level of education has not been evaluated in this manner. Although the increase in social liberation was largest in smokers, this result should be interpreted cautiously because of the small number of smokers (n = 23) in the study. Contrary to expectations, neither the behavioral or cognitive processes of change measures predicted pedometer-defined PA. Two behavioral and 1 cognitive process of change measure predicted changes in fitness, although the effects were small (likely because of the lack of an overall postintervention change in fitness). As hypothesized, changes in reinforcement management and consciousness-raising were positive predictors of fitness changes; contrary to our hypothesis, change in helping relationships was a negative predictor. Although these results generally confirm other research highlighting associations between sociodemographic, psychosocial, and behavioral variables and change in PA behavior (31), our results should be interpreted cautiously because of the lack of an overall intervention effect on fitness.

Four of the 6 social support measures exhibited significant improvements postintervention, given the emphasis on social support in HUB City Steps. Other than the negative association between baseline BMI and changes in group reliable alliance, none of the participant demographics were significant predictors of changes in social support measures. These results imply that the intervention was successful in improving social support across all HUB City Steps participants. However, group guidance, 1 of the measures that increased postintervention, was the only social support construct predictive of PA, exhibiting an unexpected negative relationship. Counter to our findings, a review of interventions in community settings found strong evidence for a positive effect of social support on increasing PA levels (32). Similar to the finding for amotivation and PA, our contradictory finding for the negative relationship between group guidance and PA may be due to the lack of a true measure of PA change from baseline for HUB City Steps participants.

This study has limitations. The lack of a randomized controlled design did not allow for a true mediation analysis of the intervention effects. During formative evaluations, community liaisons indicated that the use of a control group might alienate some community members. Furthermore, because of the close-knit nature of the targeted community and recruitment of multiple family and social group members, contamination between treatment groups would have been likely with a randomized controlled design. Political and pragmatic factors must be balanced against design rigor when conducting community-engaged interventions. Additionally, some PSC measures had unacceptable internal consistency (<0.60), bringing their reliability into question for this cohort of participants, which reduces the likelihood of finding significant results. Generalizability of the results is limited because of our predominantly African American, female cohort; other researchers also have reported difficulties recruiting and retaining African American adult men for study participation. Therefore, results with sex differences should be interpreted cautiously. As with all self-reported data, the possibility of bias in outcomes resulting from faulty recall or provision of socially desirable responses exists.

This article is among the first to address how measures of several theoretical frameworks of behavior change influence changes in PA and dietary outcomes in a multicomponent, community-based, lifestyle intervention conducted with African American adults. Positive changes in some, but not all, components of self-determination theory, processes of change, and social support predicted improvements in PA or dietary behaviors. Findings reported emphasize motivational factors on which health behavior interventions can focus. Looking beyond the global perspective of process of change (ie, behavioral vs cognitive) to specific, combined components of different processes (eg, helping relationships and social liberation) may lead to more culturally acceptable and effective interventions. Increasing participant engagement and motivation to change while decreasing attrition through improved understanding of culturally appropriate psychosocial features to target may improve the efficacy of health behavior interventions.
